# 0354. Effects of sodium nitroprusside in addition to therapeutic hypothermia after experimental cardiac arrest

**DOI:** 10.1186/2197-425X-2-S1-P20

**Published:** 2014-09-26

**Authors:** K Donadello, FS Taccone, F Su, K Hosokawa, L Gottin, J Creteur, D De Backer, J-L Vincent

**Affiliations:** Erasme University Hospital, Intensive Care Department, Brussels, Belgium; University of Verone, School of Medicine, Policlinico G.B. Rossi, Intensive Care Department, Verone, Italy

## Introduction

Sodium nitroprusside (SNP) has been shown to provide additional protective effects when combined with therapeutic hypothermia (TH) in some experimental models of cardiac arrest (CA) [1,2,3].

## Objectives

To determine whether the addition of SNP to TH has beneficial effects on the brain in a porcine model of CA.

## Methods

We studied 8 anesthetized, invasively monitored and mechanically ventilated domestic pigs, randomized into two groups (n=4): TH without or with SNP. After 3 min of untreated ventricular fibrillation, cardiopulmonary resuscitation (CPR) was started in all animals and continued until return of spontaneous circulation (ROSC); defibrillation was performed 3 minutes after the start of CPR. Hypothermia (34±1°C) was induced at the start of CPR using a rapid IV infusion of 30 mL/kg cold saline for 60 min, trans-nasal evaporative cooling (Rhinochill, Benechill Inc, USA) and surface cooling with ice packs. Cooling was maintained for 6 hours, followed by controlled slow rewarming to baseline temperature with blankets. SNP+TH animals received 3 bolus injections of 1 mg of SNP after 2, 7 and 12 minutes of CPR. Brain temperature was measured with intraparenchymal probes (Licox CC1.SB, Integra, NeuroSciences Ltd., Hampshire, UK), blood brain flow by laser Doppler (blood laser Doppler [BLD], MNP100XP, Oxyflow, Oxford Optronix, Oxford, UK) and the lactate-pyruvate ratio (LPR) was measured hourly by microdialysis (CMA20, CMA, Sweden). After left craniectomy, the microvascular network of the frontal cortex was evaluated using sidestream dark-field videomicroscopy (Microscan, MicroVision Medical, Netherlands) at baseline (T0), 1 hour after cooling induction (T1), at the end of hypothermia (T2) and after rewarming (T3). The mean flow index (MFI) and the proportion of perfused cerebral small vessels (PPV) were calculated using standard formulas.

## Results

Time to return of spontaneous circulation was similar in both groups (8 [7-23] min for TH alone and 9 [6-22] min for TH+SNP). Despite its known vasodilatory effects, there were no significant differences in measured hemodynamic parameters between the groups throughout the study period. Microvascular perfusion was significantly reduced after CA in both groups, but to a lesser extent in the TH-SNP than in the TH group. The LPR was lower in the TH-SNP than in the TH group (Result Table 1).

**Table 1:** Table 1

Time	T0	T0	T1	T1	T2	T2	T3	T3
Study Group	TH	TH-SNP	TH	TH-SNP	TH	TH-SNP	TH	TH-SNP
Heart rate, bpm	73.7 ± 9.3	73.5 ± 11.5	86.5 ± 7.6	89.5 ± 7.4	55.8 ± 11.8	55.5 ± 6.4	83.3 ± 9.8	80.6 ± 4.3
Mean arterial pressure, mmHg	113.3 ± 8.4	112.0 ± 5.4	103.3 ± 8.6	94.5 ± 8.4	86.8 ± 18.1	79.0 ± 9.4	82.8 ± 10.3	90.3 ± 3.9
PPV, %	86.8 ± 3.4	86.5 ± 2.7	39.5 ± 12.6$	45.3 ± 6.3$	39.6 ± 14.2$	49.8 ± 13.2$	56.3 ± 6.8$	65.5 ± 6.7$
MFI	2.8 ± 0.1	2.8 ± 0.1	1.9 ± 0.1$	2.0 ± 0.1$	1.7 ± 0.1$	1.9 ± 0.2$	2.3 ± 0.2$	2.7 ± 0.1*$
BLD (%/baseline)	100	100	42.1 ± 1.9$	51.3 ± 3.5$	52.8 ± 2.9$	53.6 ± 2.5$	90.7 ± 0.6$	92.5 ± 2.1$
LPR	12.5 ± 1.8	13.9 ± 4.1	16.3 ± 4.1	19.7 ± 1.7	31.7 ± 5.4$	24.3 ± 3.3$	61.1 ± 13.8$	40.5 ± 1.3*$

(*= p< 0.05 versus TH-SNP;^$^ = p< 0.05 versus T0).

## Conclusions

In this model, the cerebral microcirculation was significantly altered after CA; addition of SNP to TH attenuated the microvascular alterations and had a protective effect on brain metabolism.
